# Nebulized heparin for patients under mechanical ventilation: a conventional data meta-analysis

**DOI:** 10.1186/cc14309

**Published:** 2015-03-16

**Authors:** GJ Glas, A Serpa Neto, J Horn, MJ Schultz

**Affiliations:** 1 Academic Medical Center, Amsterdam, the Netherlands; 2 Hospital Israelita Albert Einstein, São Paulo, Brazil

## Introduction

Mechanical ventilation has the potential to induce pulmonary coagulopathy. Local treatment by nebulization of heparin could be beneficial in ventilated patients. The aim of this data metaanalysis is to determine the association between nebulization of heparin and outcome of mechanically ventilated critically ill patients.

## Methods

PubMed, Scopus, EMBASE, and Web of Science were searched for relevant articles. Articles were selected if they compared nebulization of heparin with standard care. The primary endpoint was overall mortality. Secondary endpoints included occurrence of pneumonia and number of ventilator-free days and alive at day 28.

## Results

**Figure 1:**
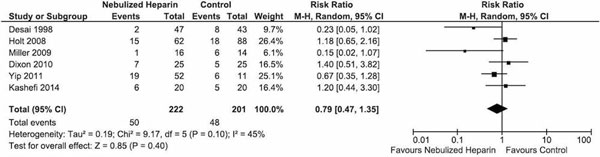
**Effect of heparin nebulization on mortality**.

Six articles were found: five retrospective cohorts with historical controls, one randomized controlled trial, covering 423 patients. Dosages of nebulized heparin varied from 30,000 to 150,000 IU/day. Fifty out of 222 patients (22.5%) receiving nebulized heparin and 48 out of 201 patients (23.9%) receiving standard care died (risk ratio (RR) 0.79 (95% CI 0.47 to 1.35)) (see Figure 1). Occurrence of pneumonia (RR 1.36 (95% CI 0.54 to 3.45); I^2 ^= 59%), and number of ventilator-free days and alive at day 28 (standardized mean difference 0.11 (95% CI -0.14 to 35); I^2^ = 0%), were not different between the two groups.

## Conclusion

Nebulization of heparin is not associated with improved outcome in mechanically ventilated critically ill patients. This metaanalysis is limited by methodological problems in most included studies. Only one randomized controlled trial could be included. Also, most patients in the meta-analyzed studies suffered from inhalation trauma, and heparin dosages differed widely.

